# On the Use of Proteine in Scrofula

**Published:** 1853-11

**Authors:** J. Taylor


### RECORD OF MEDICAL SCIENCE.

### PATHOLOGY AND PRACTICE OF MEDICINE.

*On the use of Proteine in Scrofula.* By J. TAYLOR, ESQ., L. A. C. —It was asserted in the year 1849, in your "Answers to Correspondents," that there were no remedial properties in proteine. *Nous verrons.* The same might be said of "milk," and yet, in some cases, this simple agent affords us very conspicuous evidence of its therapeutic quality, as the following case will show :—

Mr. W——, aged thirty-three, a tall and well proportioned man, with blue eyes and light hair, had always enjoyed excellent health; he had lately invested a considerable sum of money as a coach proprietor, and being desirous of turning the affair to the greatest possible advantage, became the driver of his own coach, took long journeys, and was very irregular in his habits; says his principal drink has been old ale, which he has not taken in inordinate quantity, and although not intemperate, had evidently been taxing his energies too heavily. He soon became the subject of dyspepsia, and amongst its various Protean forms was a constant vomiting of food after every meal, which persisted, in spite of appropriate and well-selected remedies, for more than a month, and so distressing was the irritability of the stomach, that not the smallest quantity of food could be retained for more than half an hour at a time. In this state he was compelled to relinquish his calling as coachman, having become emaciated and debilitated, and was attended by his physician and myself. He had no pain or tenderness in any part of the abdomen; no morbid appearance of the tongue; no headache or any preternatural heat of skin. There was craving for food, but no pain after taking it. Bowels costive; urinary secretion scanty, but of normal character. The persevering use of medicine for nearly three weeks appearing to be of no avail, it was determined to abandon the use of drugs altogether, and the patient was directed to take *a teaspoonful of milk* every hour and nothing else. The first day's trial of this remedy was so gratifying, that the patient exclaimed, "I know it will cure me, as I feel so comfortable after each dose." And so it did; the vomiting immediately ceased, and did not return; he continued his milk in gradually increased doses for more than a week, and then carefully resumed his usual diet. His recovery was rapid. So much for milk. Now for proteine.

John H——, aged five years, a scrofulous boy, born of scrofulous parents, has had enlarged cervical and inguinal glands since the period of dentition; has now numerous ulcers in various parts of his body and limbs; is pale and emaciated, with defective appetite; has been taking iron and other tonics, with and without iodine in combination, during the last six weeks, without any evident improvement. Ordered, three grains of proteine, to be taken three times a day, in sugar and water. After the first week the boy was decidedly better in his general health, looked more healthy, appetite improved considerably. In a month the mother remarked "she never saw such a change;" the boy was growing plump, many of the ulcers had healed, though a few fresh ones had appeared. The dose of proteine was increased to four grains three times a day, and the ulcers to be dressed with zinc ointment.

Third month.—All the ulcers have healed except four, and when a fresh one appears it is much smaller than usual. Increase the dose of proteine to five grains three times a day.

Fourth month.—Three or four recent small ulcers still open; the boy's health so much improved that his aunt, who had not seen him for six weeks, did not know him again, and his father observed that as the boy was so much better, it would be needless to incur any further expense, and requested the medicine might be discontinued. The proteine was consequently omitted for a fortnight, and the little patient's health was observed to decline. The parents therefore requested the medicine to be resumed, and his health rapidly improved again. The proteine was continued for about two months longer, in not more than five-grain doses, twice, and sometimes only once, a day, embracing altogether a period of somewhat more than six months, when my little patient was observed to be quite well. It may be remarked that this solitary case proves nothing. I could produce others, but the following may suffice:—

Jane B——, aged two years, an emaciated, strumous child, with tumid abdomen and enlarged cervical glands, and numerous ill-conditioned ulcers on the loins, nates, thighs, legs, and arms, has evinced symptoms of mesenteric disease ever since weaning, at nine months old; has been under the care of a surgeon for a month, and during that time has been gradually getting worse. Ordered zinc ointment, and occasionally a poultice of equal parts of linseed-meal and wheaten flour to be applied to the ulcerated parts; and to take proteine, two grains, soda exsiccata, one grain three times a day, in sugar and water.

First week.—The skin has become cleaner and more healthy, and some of the ulcers have healed; several that are now open display in a very remarkable manner the appearance of softened tubercles; the child looks more lively; bowels regular; appetite better; takes beef-tea twice a day, and milk night and morning. To have mutton for dinner.

Second week.—Greatly improved in every respect; has begun to run about again, which she has been incapable of doing for the last six weeks; nearly all the ulcers have healed; abdomen smaller; has gained flesh; appetite excellent; bowels regular; sleeps well. Ordered the proteine to be continued in doses of three grains, soda exsiccata, one grain twice a day. The mother did not bring her again, but I saw her on passing the house a month afterwards, running about in excellent health and spirits.—*London Lancet.*

